# In Vitro Inhibition of Xanthine Oxidase Purified from Arthritis Serum Patients by Nanocurcumin and Artemisinin Active Compounds

**DOI:** 10.3390/molecules28135124

**Published:** 2023-06-29

**Authors:** Waseem Yousif M. AL-dulaimy, Asmaa A. Hussein, Mohammed Asaad Mahdi, Mohammed Kadhom

**Affiliations:** 1 Department of Chemistry, College of Science, University of Diyala, Baquba 32001, Iraq; 2 Department of Molecular and Medical Biotechnology, College of Biotechnology, Al-Nahrain University, Jadriya, Baghdad 64074, Iraq; 3 Department of Environmental Science, College of Energy and Environmental Science, Al-Karkh University of Science, Baghdad 10081, Iraq; kadhom@kus.edu.iq

**Keywords:** xanthine oxidase, curcumin, artemisinin, molecular docking, inhibition

## Abstract

Curcumin and artemisinin are commonly used in traditional East Asian medicine. In this study, we investigated the inhibitory effects of these active compounds on xanthine oxidase (XO) using allopurinol as a control. XO was purified from the serum of arthritis patients through ammonium sulfate precipitation (65%) and ion exchange chromatography on diethylaminoethyl (DEAE)-cellulose. The specific activity of the purified enzyme was 32.5 U/mg protein, resulting in a 7-fold purification with a yield of 66.8%. Molecular docking analysis revealed that curcumin had the strongest interaction energy with XO, with a binding energy of −9.28 kcal/mol. The amino acid residues Thr1077, Gln762, Phe914, Ala1078, Val1011, Glu1194, and Ala1079 were located closer to the binding site of curcumin than artemisinin, which had a binding energy of −7.2 kcal/mol. In vitro inhibition assays were performed using nanocurcumin and artemisinin at concentrations of 5, 10, 15, 20, and 25 µg/mL. Curcumin inhibited enzyme activity by 67–91%, while artemisinin had a lower inhibition ratio, which ranged from 40–70% compared to allopurinol as a control.

## 1. Introduction

Xanthine oxidoreductase (XOR) is a widely studied enzyme found in various species ranging from bacteria to humans. Despite some variations in its structure and catalytic activity among different species, XOR can be categorized into two types: xanthine dehydrogenase (XDH, EC 1.1.1.204) and xanthine oxidase (XO, EC 1.1.3.22) [1,2,3]. These two forms represent distinct enzymatic states of the same gene product. In mammals, XOR is initially synthesized as XDH and then converted into XO through an irreversible post-translational modification triggered by different stimuli [4]. Under normal conditions, XOR levels in circulation are naturally low but can significantly increase in response to various diseases, particularly those affecting the liver [5]. XOR has been implicated in detrimental effects, particularly in different types of ischemic-reperfusion (IR) injury, due to the generation of reactive oxygen/nitrogen species [6,7,8]. Hyperuricemia, primarily caused by XOR-mediated overproduction or renal tubular disorders and underexcretion of uric acid, is the main pathology associated with XOR activity. Although synthetic analogs are commonly used for gout treatment, the search for new and potent XO inhibitors with minimal side effects remains crucial for the management of gout and other XOR-related diseases [9]. In this study, we aimed to investigate the potential of natural XO inhibitors, namely nanocurcumin and artemisinin.

The molecular mechanisms involved in the regulation of inflammation, immunity, oxidative stress, and cell death play a crucial role in the development of chronic kidney diseases (CKD) and acute kidney injury (AKI) [10]. CKD and AKI lead to a decline in kidney function and are associated with high morbidity and mortality rates. Natural products have been studied extensively for their potential in treating kidney diseases due to their traditional use and multi-target properties [11].

The utilization of natural compounds in drug products has been a common practice for the treatment of various diseases [12,13]. One such compound is curcumin, a polyphenol and the primary curcuminoid found in turmeric, which is derived from the Curcuma longa plant and has a vibrant yellow color [14,15]. Curcumin has demonstrated numerous benefits, including antidiabetic, anticancer, antioxidant, and anti-inflammatory effects [16,17]. It has also been shown to inhibit the production of pro-inflammatory cytokines such as TNF-α and IL-6 in certain models [18,19]. Based on these characteristics, it was hypothesized that curcumin may reduce pro-inflammatory cytokine levels in patients undergoing hemodialysis [20]. Nanocurcumin, a curcuminoid-containing nanomicelle structure, exhibits a higher bioavailability compared to its native compound, making it more soluble and absorbable [21].

According to studies, nanocurcumin has been shown to possess higher antioxidant, anti-inflammatory, antiproliferative, and neuroprotective properties compared to normal curcumin [22]. Nanocarriers used for delivering nanocurcumin include liposomes, micelles, magnetic nanoparticles, dendrimers, and polymeric or lipid-based carriers [23].

**Scheme 1:**
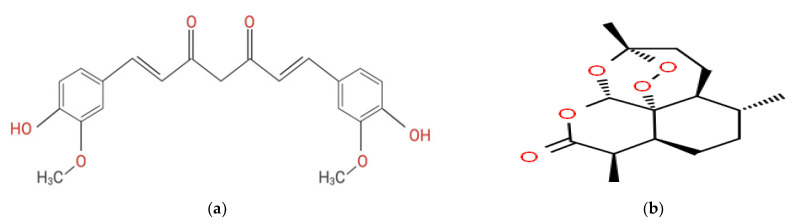
Chemical structure of (**a**) curcumin and (**b**) artemisinin.

Artemisinin (ARS) is a potent compound with a molecular weight of 282 derived from Artemisia annua L., a traditional Chinese medicinal plant. It was first discovered in 1972 by Chinese researchers. The chemical structure of artemisinin consists of a sesquiterpene lactone with peroxide bridges, which have been proven to exhibit superior antimalarial effects [24,25]. When exposed to heme or free iron, the endoperoxide bridge in artemisinin generates carbon-centered free species and reactive oxygen radicals that can directly target and kill parasites [26]. The chemical structures of curcumin and artemisinin are shown in Scheme 1. Artemisinin is considered a preferred drug for treating malaria due to its ability to specifically target and eliminate plasmodium-infected red blood cells without causing harm to healthy cells. It has been found to be more effective than other antimalarial drugs, including chloroquine (CQ) and hydroxychloroquine (HCQ) [27,28]. When taken orally, artemisinin exhibits a rapid onset of action and is readily absorbed by the gastrointestinal tract. The drug has a half-life ranging from 2 to 5 h and is primarily distributed in the liver, kidney, and bile. Approximately 80% of the drug is eliminated from the body within 24 h through urine and feces [29].

In this work, the serum of arthritis patients was used to obtain XO through an ion exchange chromatography process. The resulting purified enzyme displayed a specific activity of 32.5 U/mg protein, leading to a 7-fold purification and a yield of 66.8%. Molecular docking analysis revealed that nanocurcumin had a high interaction energy with XO (−9.28 kcal/mol) and was found to bind closer to specific amino-acid residues compared to artemisinin (−7.2 kcal/mol). In vitro inhibition assays using nanocurcumin and artemisinin indicated that curcumin showed a significant inhibition (67–91%), whereas artemisinin exhibited 40–70% compared to the control, allopurinol.

## 2. Results and Discussion

### 2.1. Nanocurcumin Characterizations 

**Figure 1:**
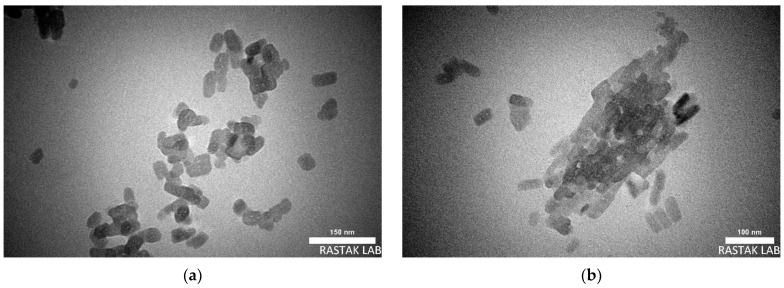
Different views of the nanocurcumin of scales, (**a**) 150 nm and (**b**) 100 nm.

Figure 1 displays images of nanocurcumin at various magnifications, revealing its capsule-shaped structure with a length range of 50–80 nm and a diameter of 15–20 nm. The small size of the particles results in a significant contact area, enhancing their reactivity and impact on the target. 

**Figure 2:**
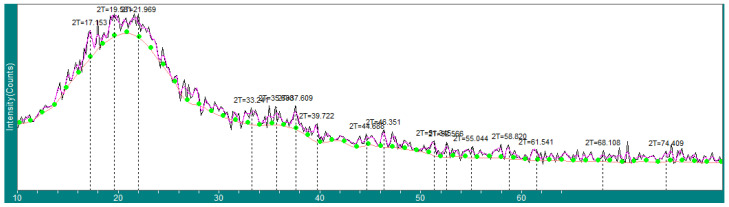
XRD test for the nanocurcumin.

Figure 2 presents the XRD examination of nanocurcumin, showcasing distinct peaks. The corresponding 2θ values were recorded as follows: 17.153, 19.581, 21.969, 33.241, 35.688, 37.609, 39.722, 44.688, 46.351, 51.345, 52.566, 55.044, 58.82, 61.541, 68.108, and 74.409. Upon comparison with reference data, it becomes apparent that the obtained peaks closely resemble those of both curcumin I and curcumin II, with a slight preference towards curcumin I. It is worth noting that curcumin I exhibits a higher melting point, lower solubility in organic solvents, and more complex XRD diffraction pattern with numerous peaks when compared to curcumin II. However, curcumin II is more thermodynamically stable and often represents the predominant crystal form in commercial turmeric extracts [30].

### 2.2. Purification of Xanthine Oxidase from Arthritis Patient’s Serum

**Figure 3:**
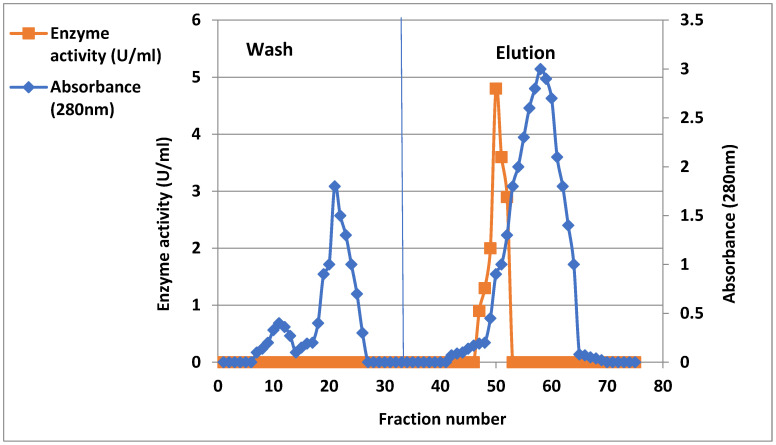
The DEAE-cellulose column chromatography (3 × 25 cm) of Xanthine oxidase purified from arthritis patients’ sera, which was eluted with 0.05 M potassium phosphate buffer at pH 7.5 using a 0.1–1 M NaCl gradient concentration at a flow rate of 30 mL/h.

Table 1 outlines the typical purification steps employed to isolate the XO enzyme from arthritis patient sera. The specific activity of the enzyme was determined to be 16 U/mg protein following 60% ammonium sulfate precipitation. However, the specific activity notably increased to 32.5 U/mg protein after elution on DEAE-cellulose, as depicted in Figure 3. This elution resulted in a single peak encompassing the majority of XO activity. During the purification process, a significant observation was made, as only one peak appeared, demonstrating the highest enzymatic activity. This peak was accompanied by a decrease in protein concentration and a concurrent increase in specific activity. This critical finding allowed for the collection of these active fractions, which were subsequently pooled and utilized for further analysis. Notably, this purification step resulted in a 7-fold purification compared to the initial crude extract, with a yield of 66.8%, as illustrated in Table 1.

The isolation of Xanthine oxidase (XO), which is a widely distributed enzyme found in various species, involves extracting the enzyme from diverse sources, such as bacteria, milk, and different animal organs. Subsequently, purification is carried out to obtain a purified form of the enzyme from the crude extract [31]. Researchers have employed various chromatographic processes and organic solvents for this purpose [32]. 

In a specific case, Buffalo liver xanthine oxidase (BLXO) was isolated to homogeneity using chromatography and acetone precipitation methods on Sephacryl S-300 and DEAE-cellulose columns. The specific activity of the purified enzyme, measured in units per milligram of protein, was determined to be 7.2 units/mg. This represents a significant increase in purity, with a 31.3-fold improvement compared to the initial crude extract [33].

### 2.3. Docking Study for Enzyme Inhibitors

Molecular enzyme docking studies were conducted to investigate the interaction between xanthine oxidase and nanocurcumin, as well as artemisinin. The findings revealed that the binding energy of nanocurcumin was determined to be −9.28 kcal/mol, while artemisinin exhibited a binding energy of −7.2 kcal/mol. Based on these results, nanocurcumin exhibited the lowest S-value, indicating the strongest interaction with xanthine oxidase.

**Figure 4:**
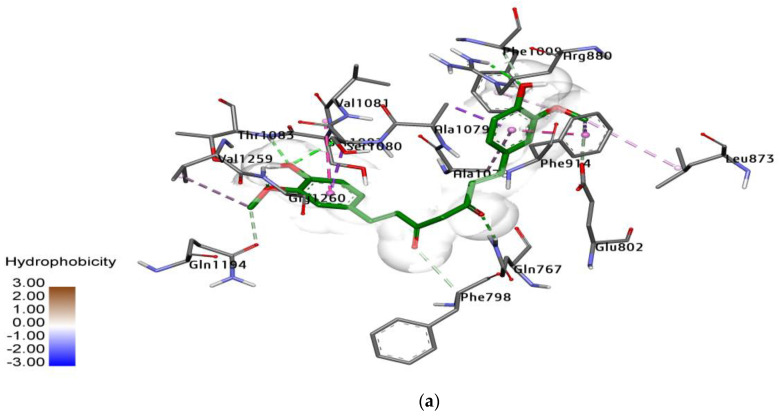

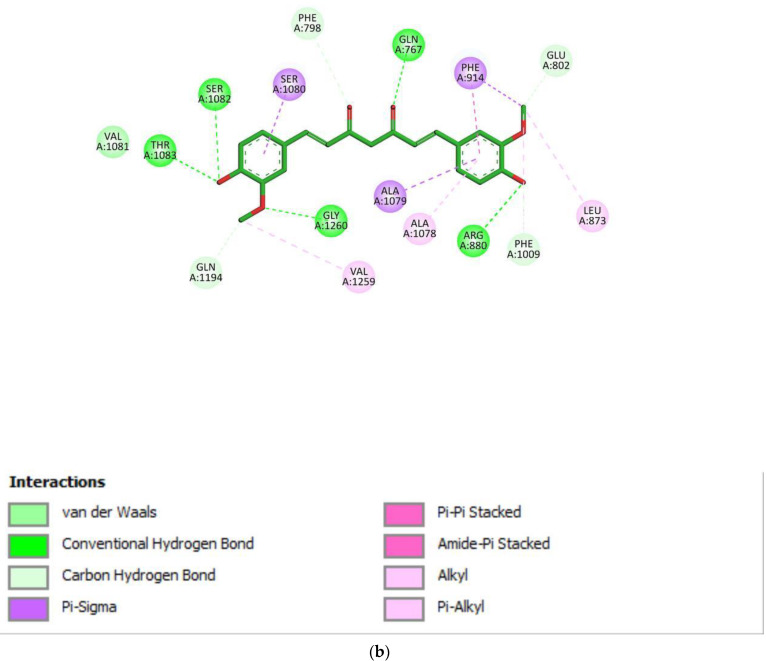
Simulation of binding nanocurcumin with Xanthine Oxidase: (**a**) 3D and (**b**) 2D views.

Figure 4 illustrates the amino-acid residues in proximity to the binding site of curcumin. These residues include Thr1077, Gln762, Phe914, Ala1078, Val1011, Glu1194, and Ala1079. The close proximity of these residues to the binding site suggests their potential involvement in the interaction between nanocurcumin and xanthine oxidase.

The principle behind molecular docking is to predict the ability of a ligand (active compound) to bind to an enzyme (receptor) and form a stable complex. This technique offers several advantages in drug design, including a reduced time and cost compared to experimental methods [34].

In a previous study by Sumirtanurdin et al., molecular docking was employed to investigate the interaction between ligands, such as curcumin and its derivatives (korkumod 23 and 24), and the target receptor Cyclin-Dependent Kinase 2 (CDK2). The binding energies were reported to be −7.80 kcal/mol, −9.15 kcal/mol, and −9.36 kcal/mol, respectively. These values indicate the strength of the interactions between the ligands and the CDK2 receptor [35].

Furthermore, in another study [15], it was mentioned that inhibitors with a low S-value were classified as having a high binding affinity for protein receptors. These inhibitors are preferred due to their effective occupation of the active site, leading to the efficient blocking of receptor protein function.

The S-value serves as an indicator of the ligand’s ability to interact with the protein receptor and its potential as a potent inhibitor

### 2.4. Xanthine Oxidase Inhibitory Activity of Nanocurcumin and Artemisinin

The impact of xanthine oxidase inhibition on uric acid production was investigated, and the active compounds exhibited inhibitory activity against xanthine oxidase at all tested concentrations. Table 2 illustrates the percentage of enzyme activity inhibition for nanocurcumin and artemisinin. Nanocurcumin demonstrated 91% inhibition at a concentration of 25 µg/mL, while artemisinin inhibited 70% of enzyme activity. The reduction in uric acid production is attributed to the inhibition of xanthine oxidase, an enzyme responsible for the conversion of xanthine to uric acid. The inhibitory effects of nanocurcumin and artemisinin on xanthine oxidase activity contribute to a decrease in uric acid production. All values in Table 2 were statistically analyzed using SPSS software, and the standard deviation was calculated.

Xanthine oxidase inhibitors (XOIs) play a significant role in the treatment of hyperuricemia and gout by reducing the levels of circulating uric acid and alleviating vascular oxidative stress associated with elevated uric acid levels. The main function of XOIs is to inhibit the biosynthesis of uric acid from purine in the body. In addition to pharmaceutical XOIs, various herbs and their extracts have been traditionally used for the treatment of conditions caused by increased xanthine oxidase activity. These natural remedies have shown potential in modulating xanthine oxidase activity and may offer alternative options for managing hyperuricemia and related disorders [36]. Turmeric, scientifically known as Curcuma longa, is among the plants that have been utilized for thousands of years in the prevention and treatment of various chronic diseases [37]. Turmeric and similar plants are known to be rich sources of flavonoids and other active compounds that exhibit potent xanthine oxidase inhibitory activity [38]. These substances have shown positive results in lowering uric acid levels and alleviating the symptoms of diseases, including hyperuricemia and gout. The use of xanthine oxidase inhibitors derived from plants, such as turmeric and other compounds, offers an all-natural and potentially successful method of treating these issues.

## 3. Materials and Methods

### 3.1. Chemicals

Nanocurcumin and artesunate were obtained from Sigma-Aldrich(St. Louis, MO, USA) with purities of 99% and 98%, respectively. Curcumin has a chemical formula of HOC_6_H_3_(OCH_3_)CH=CHCO]_2_CH_2_, while artesunate has a chemical formula of C_19_H_28_O_8_ and was in microsize. Additionally, we procured a range of high-quality chemicals for our study from reputable suppliers, in order to process the investigation. Here, Odium nitrate, DMSO, MTT stain, EDTA, RPMI 1640 media, RPMI media, trypsin, fetal bovine serum, ascorbic acid, sodium chloride, and sodium bicarbonate were obtained from Sigma-Aldrich (MO, USA). These chemicals are essential for various experimental procedures and analytical assays conducted during our research. Additionally, we sourced Tris-HCl, bovine serum albumin (BSA), Coomassie brilliant blue G-250, DEAE-cellulose, sodium chloride, aluminum chloride, hydrochloric acid, and rutin from BDH (Dubai, United Arab Emirates). These high-quality reagents were carefully selected to ensure accurate and reliable results for our study.

### 3.2. Characterization Instrumentations

Curcumin, which exhibited a higher impact compared to artesunate, was characterized using transmission electron microscopy (TEM) and X-ray diffraction (XRD). TEM is a technique used to visualize and analyze the size, shape, distribution, and crystal structure of nanoparticles. In this study, TEM images were acquired using the EM 208S (Philips) device. To prepare the samples for TEM, a mixture of nanoparticles (NPs) and ethanol was placed onto a carbon-coated copper grid. The solution was then air-dried at room temperature. This sample preparation method allows for the visualization of the nanoparticles under TEM.

TEM works by directing a beam of electrons through the sample, which interacts with the nanoparticles and produces an image that can be magnified and analyzed. By examining the TEM images, detailed information about the size, shape, and distribution of the nanoparticles can be obtained, providing insights into the physical characteristics of curcumin in this study. Additionally, XRD analysis can be performed to further investigate the crystal structure of curcumin and provide complementary information about its molecular arrangement.

In X-ray diffraction (XRD), a beam of X-rays is directed at a sample, and the interaction of the X-rays with the atoms in the sample produces a diffraction pattern that can be detected and analyzed. The diffraction pattern provides valuable information about the crystal structure of the sample, including the lattice spacing, the orientation of crystal planes, and size and shape of the unit cell. The angle between the incident beam and the diffracted beam is known as the diffraction angle (theta, θ). In XRD, the diffraction angle is typically measured as 2θ, which is twice the angle between the incident and diffracted beams. By measuring the 2θ values for a series of diffraction peaks from a sample, it is possible to determine the crystal structure, lattice parameters, and other properties of the material.

In our study, the XRD measurements were conducted using the X’Pert Pro MRD apparatus from PANalytical. This XRD instrument is capable of analyzing the diffraction pattern produced by the X-rays interacting with the curcumin sample. The obtained diffraction data can be further analyzed to gain insights into the crystal structure and other relevant characteristics of curcumin.

### 3.3. Extraction and Purification of XO from Serum

Xanthine oxidase was extracted and purified from the serum of Iraqi patients suffering from arthritis using a two-step purification technique, as described by [39].

In the first step, solid-phase ammonium sulfate was gently added to 50 mL of the crude enzyme to achieve a 65% saturation ratio. The mixture was then centrifuged for 20 min at 6000 rpm, and the resulting precipitate was dissolved in an appropriate amount of 0.05 M phosphate buffer. In the second step, the precipitate obtained from the previous step was dialyzed against the aforementioned buffer for 24 h under cooling conditions (4 °C), using a dialysis tube with a 3500 Mw cutoff. Enzyme activity and protein concentration were determined.

In the third step, the enzyme solution obtained from the previous step was applied to a 3 × 25 cm DEAE-cellulose column, which had been pre-equilibrated with potassium phosphate buffer (0.05 M, pH 7.5). The column was rinsed with the same buffer, and XO was subjected to a 0.1–1 M NaCl concentration gradient at a flow rate of 30 mL/h.

### 3.4. Xanthine Oxidase Activity Assay

The xanthine oxidase activity was determined using the method outlined in [40]. The absorbance at 283 nm was measured using a UV-visible spectrophotometer. Enzymatic activity was determined based on the production of uric acid resulting from the oxidation of xanthine. The unit of enzyme activity was defined as the amount of enzyme that produced uric acid per minute under the specified assay conditions. Protein concentration was estimated using a previously established protocol [41], with bovine serum albumin serving as the standard.

### 3.5. Molecular Docking of Curcumin and Artemisinin 

The protein-ligand binding affinity was assessed using the Molecules Operator Environment (MOE) software, following the procedure described in [42]. The Xanthine oxidase protein was obtained from the Protein Data Bank (PDB ID: 3bdj), and its structure was optimized by removing water molecules, adding hydrogen atoms, adding missing side chains, and minimizing its energy.

The structures of the inhibitors were also optimized through energy minimization. The binding affinity was evaluated using a statistical value known as the S-value, which was calculated based on the identification of hydrogen bonds, salt bridges, solvation effects, and hydrophobic interactions [43].

### 3.6. Xanthine Oxidase Inhibitory Activity

The XO-hindering activity under aerobic conditions was assessed using a modified version of the method described in [16]. The reaction mixture consisted of 50 μL of a test solution ranging from 6.25 to 100.00 μg/mL, 30 μL of 70 mM phosphate buffer with a pH of 7.5, and 30 μL of an enzyme solution in 70 mM phosphate buffer with a pH of 7.5, prepared immediately prior to use at a concentration of 0.01 units/mL. The mixture was pre-incubated for 15 min at 25 °C, and the reaction was initiated by adding 60 μL of a substrate solution containing 150 μM xanthine in buffer. The reaction mixture was then incubated for 30 min at 25 °C. To stop the reaction, 25 μL of 1 N hydrochloric acid (HCl) was added, and the absorbance was measured at 290 nm. The inhibition ratio of xanthine oxidase activity was calculated using the following equation:

(1) $$\mathrm{Inhibitory}\;\mathrm{percentage}\;(\%)=\frac{\Delta OD_{control}-\Delta OD_{sample}}{\Delta OD_{control}}\times 100$$

## 4. Conclusions

The XO protein was retrieved from the Protein Data Bank, and its structure was optimized by removing water molecules, adding hydrogen atoms, adding missing side chains, and minimizing its energy. The structures of the inhibitors were also optimized through energy minimization. The binding affinity was evaluated using a statistical value (S-value), which was estimated by identifying hydrogen bonds, salt bridges, solvation effects, and hydrophobic interactions. The XO was extracted and purified from the serum of Iraqi patients suffering from arthritis using a two-step purification technique. The impact of nanocurcumin and artesunate on enzyme activity was characterized via TEM and XRD techniques. Nanocurcumin and artemisinin were used in in vitro inhibitory experiments at doses of 5, 10, 15, 20, and 25 g/mL. The findings demonstrated that nanocurcumin had a larger effect than artesunate, using allopurinol as a control. This research paves the way for further studying nanosize natural materials and utilizing them in therapy applications.

## Figures and Tables

**Table 1:** Xanthine oxidase purification steps from sera of arthritis patients.

Purification Step	Volume (mL)	Enzyme Activity (U/mL)	Protein Concentration (mg/mL)	Specific Activity (U/mg)	Total Activity (U)	Purification (Folds)	Yield (%)
Crude enzyme	50	1.4	0.3	4.6	70	1	100
Ammonium sulfate precipitation 65%	13	4	0.25	16	52	3.4	74.2
DEAE-cellulose	18	2.6	0.08	32.5	46.8	7	66.8

**Table 2:** Active compounds as xanthine oxidase inhibitors.

Compound	Xanthine Oxidase Inhibition %				
	5 (µg/mL)	10 (µg/mL)	15 (µg/mL)	20 (µg/mL)	25 (µg/mL)
Nano-Curcumin	67.61 ± 1.52	84.01 ± 1.49	87.32 ± 2.08	89.01 ± 2.64	91.12 ± 2.34
Artemisinin	40.61 ± 1.15	50.32 ± 3.21	56.01 ± 2.31	59.21 ± 2.11	68.71 ± 2.56
Allopurinol (Control)	46.33 ± 6.50	55.12 ± 5.13	78.37 ± 3.15	83.29 ± 2.45	86.01 ± 4.33

## Data Availability

The provided data can be found at the Diyala Teching Hospital.

## Sample Availability

Samples of the compounds are not available from authors.
